# Family support in treatment of patients diagnosed with schizophrenia: a case report

**DOI:** 10.1192/j.eurpsy.2022.2062

**Published:** 2022-09-01

**Authors:** A. Jambrosic Sakoman, D. Bosnjak Kuharic, I. Zegura

**Affiliations:** University Psychiatric Hospital Vrapče, Department For Psychotic Disorders, Zagreb, Croatia

**Keywords:** negative symptoms, schizophrénia, Family, Treatment

## Abstract

**Introduction:**

Schizophrenia is often accompanied by functional deterioration which can impede a person‛s everyday activities. Families are an important part in providing care for their ailed member‚ but often lack resources to deal with different challenges included in such care. Mental health professionals treating such patients sometimes neglect the importance of including their families‚ or primary caretaker in the treatment plan.

**Objectives:**

We will present two cases of patients diagnosed with schizophrenia with prominently negative symptoms and the challenges met by their caretakers and mental health professionals in the treatment.

**Methods:**

Patient history‚ psychiatric evaluation, psychiatric treatment‚ and the role of the family will be presented.

**Results:**

21 years old patient came with his mother after years of not meeting the expected level of functioning. He was misdiagnosed for a couple of years. His treatment was impeded by loss in the family‚ which also affected both him and his mother. Inability to include her earlier in his treatment became a challenge. Second patient has been treated for schizophrenia with dominant negative symptoms‚ inconsistently‚ for ten years. Her family became more involved in her treatment only after she presented with positive symptoms. Their involvement was important as it resulted in patient‛s better compliance.

**Conclusions:**

Supporting a family member diagnosed with schizophrenia can be overwhelming for the families. Including family members in the treatment early on, can be beneficial both for the patient and the family.

**Disclosure:**

No significant relationships.

